# IUC26661-86 Real-world outcomes in patients with Metastatic Hormone-Sensitive Prostate Cancer treated with androgen deprivation therapy and Apalutamide - A single centre study

**DOI:** 10.1093/oncolo/oyag286.012

**Published:** 2026-08-21

**Authors:** K Marchon, S Brown, K Swaminathan, S Adeyemi, G Kapur

**Affiliations:** Norfolk and Norwich University Hospital, Norwich - United Kingdom; Norfolk and Norwich University Hospital, Norwich - United Kingdom; Norfolk and Norwich University Hospital, Norwich - United Kingdom; Norfolk and Norwich University Hospital, Norwich - United Kingdom; Norfolk and Norwich University Hospital, Norwich - United Kingdom

## Abstract

**Background:**

Apalutamide, in combination with androgen deprivation therapy (ADT), is used to treat patients with metastatic hormone-sensitive prostate cancer (mHSPC). However, real-world evidence describing its effectiveness and tolerability remains limited. This study aimed to assess outcomes in mHSPC patients treated with apalutamide at a single centre. The primary objective was to evaluate treatment-related adverse events. Secondary objectives include overall survival, prostate-specific antigen (PSA) and radiographic progression-free survival.

**Methods:**

A retrospective analysis of 167 patients with mHSPC on ADT commenced on apalutamide between August 2021 and January 2024.

**Results:**

The median age was 73 years (IQR: 67-76), and 93% had an ECOG performance status ≤ 1. Prior radical treatment, including surgery and radiotherapy, had been received by 21% of patients. The median PSA at the time of diagnosis of metastatic disease was 46.6µg/L (IQR: 14.2-211.95). Bone, lymph node and visceral metastases were present in 79%, 68% and 11% of patients, respectively. Low-volume disease was present in 66% of patients and high-volume disease in 34%. Median follow-up was 34 months. The percentage of patients with PSA and radiographic progression-free survival at 36 months was 75.2% and 75.3%, respectively. Overall survival at 36 months was 72.5%. Overall, 80 patients (48%) experienced at least one treatment-related adverse event. The most frequently reported adverse events were fatigue (18.5%), hot flushes (14%), rash (9.5%), cardiovascular (6%), musculoskeletal (6%), cerebrovascular (3%) and other adverse events (9%). Grade ≥3 adverse events occurred in 27 patients (16%), most commonly severe rash and cardiovascular events. Permanent treatment discontinuation, dose reductions and treatment interruptions due to adverse events occurred in 13.8%, 15.6%, 7.2% patients, respectively.

**Conclusions:**

Apalutamide combined with ADT demonstrated durable disease control comparable to clinical trial outcomes in the real-world setting. While the overall incidence of reported adverse events in our study was lower than in the TITAN trial (48% vs 96.8%), serious adverse events resulting in treatment discontinuation occurred more frequently (13.8% vs 8%). These findings highlight the differences between clinical trials and real-world populations and may reflect the retrospective nature of the study and under-reporting of lower-grade toxicities in routine clinical practice.

